# Survival of Skin Graft between Transgenic Cloned Dogs and Non-Transgenic Cloned Dogs

**DOI:** 10.1371/journal.pone.0108330

**Published:** 2014-11-05

**Authors:** Geon A Kim, Hyun Ju Oh, Min Jung Kim, Young Kwang Jo, Jin Choi, Jung Eun Park, Eun Jung Park, Sang Hyun Lim, Byung Il Yoon, Sung Keun Kang, Goo Jang, Byeong Chun Lee

**Affiliations:** 1 Department of Theriogenology & Biotechnology, College of Veterinary Medicine, Seoul National University, Seoul, Republic of Korea; 2 Central Research Institutes, K-stem cell, Seoul, Republic of Korea; 3 Laboratory of Histology and Molecular Pathogenesis, College of Veterinary Medicine, Kangwon National University, Chuncheon, Gangwon-do, Republic of Korea; INRA, France

## Abstract

Whereas it has been assumed that genetically modified tissues or cells derived from somatic cell nuclear transfer (SCNT) should be accepted by a host of the same species, their immune compatibility has not been extensively explored. To identify acceptance of SCNT-derived cells or tissues, skin grafts were performed between cloned dogs that were identical except for their mitochondrial DNA (mtDNA) haplotypes and foreign gene. We showed here that differences in mtDNA haplotypes and genetic modification did not elicit immune responses in these dogs: 1) skin tissues from genetically-modified cloned dogs were successfully transplanted into genetically-modified cloned dogs with different mtDNA haplotype under three successive grafts over 63 days; and 2) non-transgenic cloned tissues were accepted into transgenic cloned syngeneic recipients with different mtDNA haplotypes and vice versa under two successive grafts over 63 days. In addition, expression of the inserted gene was maintained, being functional without eliciting graft rejection. In conclusion, these results show that transplanting genetically-modified tissues into normal, syngeneic or genetically-modified recipient dogs with different mtDNA haplotypes do not elicit skin graft rejection or affect expression of the inserted gene. Therefore, therapeutically valuable tissue derived from SCNT with genetic modification might be used safely in clinical applications for patients with diseased tissues.

## Introduction

Somatic cell nuclear transfer (SCNT) produces genetically identical cloned animals [1]. Moreover, canine SCNT combined with transgenic technologies can make genetically identical cloned dogs with functional genetic modifications that could be used for gene therapy [2]. For example, transgenic cloned dogs could be used in replacement of diseased (malfunctioning/worn out) organs. However, tissues derived from transgenic cloned dogs, reprogrammed from somatic cells with enucleated oocytes, had not yet investigated whether they are immunologically identical tissues or cell sources of transplantation. Especially, effects of red fluorescent protein (RFP) expression using genetically identical animal models derived from SCNT have not been described and this is a critical subject since RFP has been used as a potential marker for clinical trials of gene therapy [3]–[5].

In addition, SCNT uses oocytes from animals unrelated to the prospective transplant recipient, oocyte-derived mitochondrial DNA (mtDNA) derived antigen could lead to rejection problems in kidney transplant [6] or not in skin transplant [7], [8]. Although tissues derived from SCNT, using the recipient’s somatic cells as nuclear donors, provide identical genetics, the absence of immune rejection has not yet been confirmed in cloned dogs.

To our knowledge, no previous report has mentioned *in vivo* skin immune responses against tissue expressing foreign gene or the capable effects of mitochondrial derived minor antigen in cloned animals. Here, we firstly evaluated the anti-foreign gene or minor antigen derived immune responses in cloned dogs with the following design: (1) for investigation of mtDNA derived antigen compatibility, skin graft was performed between transgenic cloned dogs with different mtDNA haplotypes; (2) furthermore, skin graft was also performed between transgenic cloned dogs and non-transgenic cloned dogs for examination of immunogenicity of foreign gene.

## Materials and Methods

### 1. Animals

Two genetically identical cloned female beagles (C1, C2) were generated by SCNT using a beagle fetal fibroblast cell line (BF3) described in a previously study [9]. Transgenic cloned female beagles (R1, R2, R3 and R5) were also produced by SCNT using BF3 transfected with RFP [2].

Non-related controls (Co1, Co2) were healthy age-matched normal female beagles purchased from commercial kennels (Marshall Beijing Biotech Ltd., Beijing, China). All animals used in this study were cared for in accordance with recommendations described in “The Guide for the Care and Use of Laboratory Animals” published by the Institutional Animal Care and Use Committee (IACUC) of Seoul National University (approval number; SNU-110915-2). Dog housing facilities and the procedures performed met or exceeded the standards established by the Committee for Accreditation of Laboratory Animal Care. All surgery was performed under isoflurane anesthesia, and all efforts were made to minimize suffering.

### 2. DNA extractions and PCR reaction

Blood was collected from two control beagles and six female cloned beagles 4 years of age for DNA extractions, blood typing and blood cross-matching. Approximately 10 ml of blood were collected from the jugular vein into tubes containing EDTA as anticoagulant and used for peripheral blood mononuclear cell isolation and DNA extraction, and 3 ml of blood in plain tubes were collected to provide serum samples for antibody levels. Blood samples were kept at 38°C to maintain cell viability.

Freshly retrieved non-coagulated blood samples were mixed with RBC lysis buffer (Invitrogen, Carlsbad, CA, USA) at room temperature for 15 min. Genomic DNA was isolated according to the manufacturer’s protocol. Extracted DNA samples were stored at −30°C. DLA class I (MHC class I) and II (MHC class II) typing analysis was performed by means of PCR and sequencing. The polymorphic exon 2 and exon 3 of the DLA-88 gene was amplified using PCR primers [10]. The polymorphic exon 2 of the *DRB1, DQA* and *DQB* genes was also amplified using PCR primers [11]. For PCR, Maxime PCR PreMix kit (iNtRON Biotechnology, Inc., Gyeongi, Korea) was used. In each PCR tube, 1 µl of genomic DNA, 1 µl (10 pM/µl) of forward primer, 1 µl (10 pM/µl) of reverse primer and 17 µl of sterilized distilled water were added according to the manufacturer’s instructions. These components were then mixed and centrifuged briefly. PCR was done using a PCR machine (Biometra, Goettingen, Germany). PCR amplification was carried out for 1 cycle with denaturing at 94°C for 5 min, and subsequently for 30 cycles with denaturing at 94°C for 40 sec, annealing at 63°C (*DLA-DRB1*), 55°C (*DLA-DQA1*) and 66°C (*DLA-DQB1*) for 40 sec, extension at 72°C for 40 sec, and a final extension at 72°C for 5 min. Amplified PCR product was run on the gel by gel electrophoresis (Mupid-exu, Submarine electrophoresis system, Advance, Japan) at 100 V for 20 min. A 2% agarose gel was prepared using agarose (Invitrogen) and 1X TAE buffer. The stain (RedSafe, iNtRON Biotechnology Inc.) was used at a concentration of 2.5 µl per 50 ml of gel. After running gels, images were made under ultraviolet light. PCR product was sequenced directly using the Big Dye Terminator kit (Applied Biosystems, Foster City, CA, USA). Sequencing was performed on an automated DNA sequencer model 377 or capillary model 3110 (Applied Biosystems).

### 3. Sequencing of Mitochondrial DNA haplotype

For mitochondrial DNA analysis, the oligonucleotide primers were synthesized over the hypervariable regions (forward, 5′-CCTAAGACTTCAAGGAAGAAGC-3′; reverse, 5′-TTGACTGAATAGCACCTTGA-3′) of the complete nucleotide sequence of canine mtDNA (GenBank accession no. U96639). Isolated genomic DNA sample were dissolved in 50 ul TE buffer and used for PCR amplifications. It were performed in a 50 µl volume containing 5 µl of 10× reaction buffer containing 1.5 mM MgCl2, 0.2 mM dNTPs, 0.2 µM each primer, 1.5 U Taq DNA polymerase (Intron, Kyunggi, Korea). Starting denaturing for 1 cycle at 95°C for 3 minutes, subsequently denaturation at 94°C for 30 seconds, annealing at 57°C for 30 seconds, extension at 72°C for 30 seconds of 35 cycles, and a final extension at 72°C for 3 minutes were carried out. After purification of PCR products using a Gel Extraction Kit (Qiagen, Hilden, Germany), they were sequenced with an ABI3100 instrument (Applied Biosystems). Their identities with mtDNA were confirmed by BLAST search (http://blast.ncbi.nlm.nih.gov/).

### 4. Blood crossmatching and blood typing

Blood collection was performed from the jugular vein of all cloned dogs (R1, R2, R3, R5, C1 and C2) into an evacuated tube containing EDTA as anticoagulant. Collected samples were submitted to a commercial laboratory kit (Antech Diagnostics, Phoenix, AZ, USA). Blood type was confirmed using the tube agglutination method with antiserum; consisting of 6 types of monoclonal antibodies for canine blood typing [12].

The blood crossmatching test was done on EDTA-treated blood using the tube agglutination method. Isolated RBCs of all dogs were washed 3 times with 0.9% saline, and a 4% RBC suspension was made from the washed cells. RBC suspensions from cloned beagles (C1) were combined with equal volumes of another cloned beagle’s serum (C2) and the reverse reaction was also performed. All mixtures were incubated at 37°C for 20 min, centrifuged and then assessed for hemolysis or agglutination. Agglutination was evaluated by comparing the color of supernatant in the test tube with those of the control sample. Each sample was shaken until all red blood cells in the “button” at the bottom of the tube had become suspended. Again, the degree of RBC clumping of the test sample was compared with that of the auto-mixture of RBC and plasma. When the plasma was clear, no clumping of RBCs was detected at 400× magnification, these results were considered as negative. A positive result showed agglutination resembling stacked coins. Images were obtained using a microscope, the ProgRes Capture camera system, and the ProgRes Capture 2.6 software (JENOPTIK, Jena, Germany).

### 5. Peripheral blood mononuclear cell isolation and mixed lymphocyte reactions

Blood was collected from two control dogs and six female cloned dogs before and 10 weeks after skin graft. EDTA-treated whole blood was transferred to 50 ml conical centrifuge tubes. An equal volume of phosphate buffered solution (PBS, Gibco, Carlsbad, CA, USA) was mixed with the sample prior to the isolation process. Peripheral blood mononuclear cells (PBMC) were isolated from EDTA-treated blood using lymphocyte separation medium on a Ficoll-paque gradient (Ficoll-Paque Plus, GE Healthcare, Pittsburgh, PA, USA). Mixed lymphocyte reactions were modified from the previous reports [13]–[15]. Washed cells were diluted in culture medium (RPMI1640, Gibco) supplemented with 10% FBS to 2×10^6^ cells/ml. To stimulate proliferation of lymphocytes, PBMCs were preincubated with 2 ug/ml of phytohemagglutin for 24 h before mix reaction. Then 50 ul of this cell suspension was added into each well of a 96-well microplate except for the wells required for the blank and cultured at 37.5°C in a water-saturated atmosphere containing 5% CO_2_. Each cell combination was tested in quadruplicate in a flat-bottomed micro plate containing 0.1 ml of culture medium per well. The mixture was cultured for 5 days and then pyrimidine analogue, bromodeoxyuridine labeling reagent (Cell proliferation ELISA, Roche Applied Science, Indianapolis, IN, USA) was added and re-incubated for 24 h. After removing the labeling medium, results are expressed as absorbance units at 450 nm wavelength read by a micro plate reader, Sunrise (Tecan Sunrise, Hayward, CA, USA). Time-course kinetics was studied by harvesting on day 7 of culture.

### 6. DNA walking

For confirmation of the transgene (RFP) location, PCR was performed with a DNA Walking SpeedUP Kit (Seegene Inc., Seoul, Korea) and products were gel purified (QIAquick PCR purification kit; QIAGEN, Valencia, CA, USA), and DNA strands were directly sequenced (Macrogen, Seoul, Korea; http://www.macrogen.com) using a custom-synthesized primer (5′-TCACAGAAGTATGCCAAGCGA-3′). The sequences, except for known sequences, including primers of each product were aligned by sequence homology analysis using the Basic Local Alignment Search Tool (BLAST) at the National Center for Biotechnology Information (NCBI) GenBank (http://blast.ncbi.nlm.nih.gov/).

### 7. Skin graft

For skin graft procedures, experimental dogs were anesthetized with ketamine hydrochloride (6 mg/kg) after pretreatment with xylazine (0.05 mg/kg), and were maintained with 2% isoflurane in oxygen. A flank skin segment 1.5 cm×1.5 cm was excised from each donor dog. Simultaneously, the same sized skin piece was excised from recipient dogs, and the excised skin was grafted by suturing into the graft bed of the same region of an anesthetized recipient dog. Bandages were changed every day after surgery and the grafts were observed weekly.

For examination of effects mtDNA haplotypes differences among cloned dogs, skin grafts of three times were performed every 4 weeks between non-transgenic cloned dogs with same mtDNA haplotype and between transgenic cloned dogs with disparate mtDNA haplotypes. Accepted tissues were maintained until 9 weeks after skin graft. Biopsies of skin were performed after 63 days after first skin graft. A flank skin segment of 1^st^ graft with size of 0.5 cm×1.5 cm including donor and recipient tissue were excised for H&E staining at 5 weeks of skin graft and remnant tissue were excised for immunofluorescence imaging and western blot at later.

### 8. Histological and immunofluorescence analysis

Immuno-staining of canine skin immune cells was carried out on formaldehyde-fixed sections using a rabbit monoclonal antibody to CD3 (1∶100, ab94756, Abcam, Cambridge, MA, USA), visualized with an anti-rabbit polyclonal DyLight 488 (1∶200, ab96895, Abcam) antibody. In these sections, CD4 and CD8 cells were counterstained with a CD4 (1∶100, LS c122857, Lifespan Bioscience Inc., Seattle, WA, USA) and CD8 (1∶200, ab22505, Abcam) specific antibody detected with a DyLight 405 (1∶200, 3069-1, Abcam) and DyLight 649 (1∶200, ab98389, Abcam) coupled secondary antibody. Skin sections were also processed for assessing expression of RFP using rabbit polyclonal RFP antibody (1∶200, ab62341, Abcam) and visualized with an anti-rabbit polyclonal DyLight 488 (1∶200, ab96895, Abcam) antibody. Sections were counterstained with 4′, 6′-diamidino-2-phenylindole (DAPI).

Histology was done by fixing skin fragment in 4% neutral formalin and embedding in paraffin; sections were stained with standard hematoxylin and eosin (H&E) procedures. Fluorescent and bright field images were obtained with a Leica DMI 6000B microscope using a DFC350 camera and LAS software (Leica Microsystems Pty Ltd., North Ryde, Australia) and analyzed by a computer-assisted image analysis system (Metamorph version 6.3r2; Molecular Devices Corporation, PA, USA). To maintain a constant threshold for each image and to compensate for subtle variability of the immune-fluorescent imaging, we only counted cells that were at least 70% lighter than the average level of each positive control image after background subtraction. All image analytical procedures described above were performed blind without knowledge of the experimental scheme.

### 9. Western blot

Skin fragments of graft was excised and homogenized in PRO-PREP protein extraction solution (iNtRON Biotechnology, Inc.) using a tissue homogenizer. After measuring protein concentration using Nanodrop 2000 (Thermo fischer scientific, Seoul, Korea), equal amounts of proteins were loaded on 10% SDS-PAGE. Proteins were electrophoresed and blotted onto polyvinylidene fluoride membranes. The membranes were blocked with 5% skim milk in TBS with 0.1% Tween-20 and incubated with primary antibodies for 2 hours at room temperature. Monoclonal CD4 and CD8 antibodies were used as markers for immune rejection. Subsequently, membranes were incubated with goat anti-mouse IgG, anti-rat IgG (Pierce, Rockford, IL, USA) with horse radish peroxidase conjugation for 1 h at room temperature. Then, WEST-one™ Western blot detection system (iNtRON Biotechnology, Inc.) was added and visualized after exposing the membrane to X-ray film.

### 10. Statistical Analysis

The data of mixed lymphocyte reaction, image analysis of immunocytochemistry and western blot were analyzed using one-way ANOVA and a protected least significant different (LSD) test using general linear models to determine differences among experimental groups. Data were analyzed using GraphPad Prism software (GraphPad Software Inc., San Diego, CA, USA). Absorbance mean values were considered significantly different when the P-value was less than 0.05. The observations of mixed lymphocyte reaction among experimental groups were replicated at least 8 times.

## Results and Discussions

It has been reported that immune rejection can occur when tissues of genetically identical SCNT cloned animals were transplanted to each other, due to the tissues having different maternally-derived antigens [6], [16],[17]. Antigens derived from mtDNA in accelerated skin rejection in syngeneic rodent recipients [18], [19]. It has also been generally assumed that genetically-engineered tissues with insertion of a foreign gene could invoke immune-rejection by the recipient even in inbred mice [20]. Using embryonic stem cells derived from SCNT, the complete rescues of genetic defect with genetically-engineered cell therapy were not observed [21]. Engraftment of hematopoietic precursor cells differentiated from SCNT or induced pluripotent stem cells (iPSCs) was only successful in the absence of natural killer cells and immunogenicity of iPSCs was reported [21]–[23].

In the present study, cloned dogs produced by SCNT had different mtDNA haplotypes (Table 1), because canine SCNT used oocytes obtained from several oocyte donor dogs and the oocyte mtDNA was still present after the SCNT procedure. To examine the immunogenicity of skin tissue derived from syngeneic grafts exhibiting different mtDNA haplotypes, we initially performed *in vitro* molecular typing of dog leukocyte antigen (DLA), mixed lymphocyte reaction (MLR) and blood cross-matching using cells derived from cloned dogs with different mtDNA haplotypes (Fig. S1). Despite the different mtDNA haplotypes, they had no effects on *in vitro* immunological compatibility.

**Table 1:** Mitochondrial DNA sequences of non-transgenic cloned dog (C2) and four transgenic cloned dogs (R1, R2, R3 and R5).

Sample	Nucleotide positions																					
	15435	15483	15508	15526	15595	15611	15612	15620	15627	15632	15639	15643	15650	15652	15781	15800	15814	15815	15912	15955	16025	16083
Reference^1^	G	C	C	C	C	T	T	T	A	C	T	A	T	G	C	T	C	T	C	C	T	A
C2	G	C	C	T	T	T	C	T	A	C	G	G	T	A	C	T	C	T	C	T	T	A
R1	G	C	C	C	C	T	T	T	A	C	T	A	T	G	C	T	T	T	C	C	T	A
R2	G	C	C	C	C	T	T	T	A	C	A	A	T	G	C	T	T	T	C	C	C	A
R3	G	C	C	C	C	T	T	C	G	C	A	A	T	G	C	T	T	T	C	T	T	A
R5	G	T	C	C	C	T	T	T	G	C	A	A	T	G	C	T	T	T	C	C	T	A

GenBank accession number :U96639 (Kim et al., 1998).

**Figure 1:**
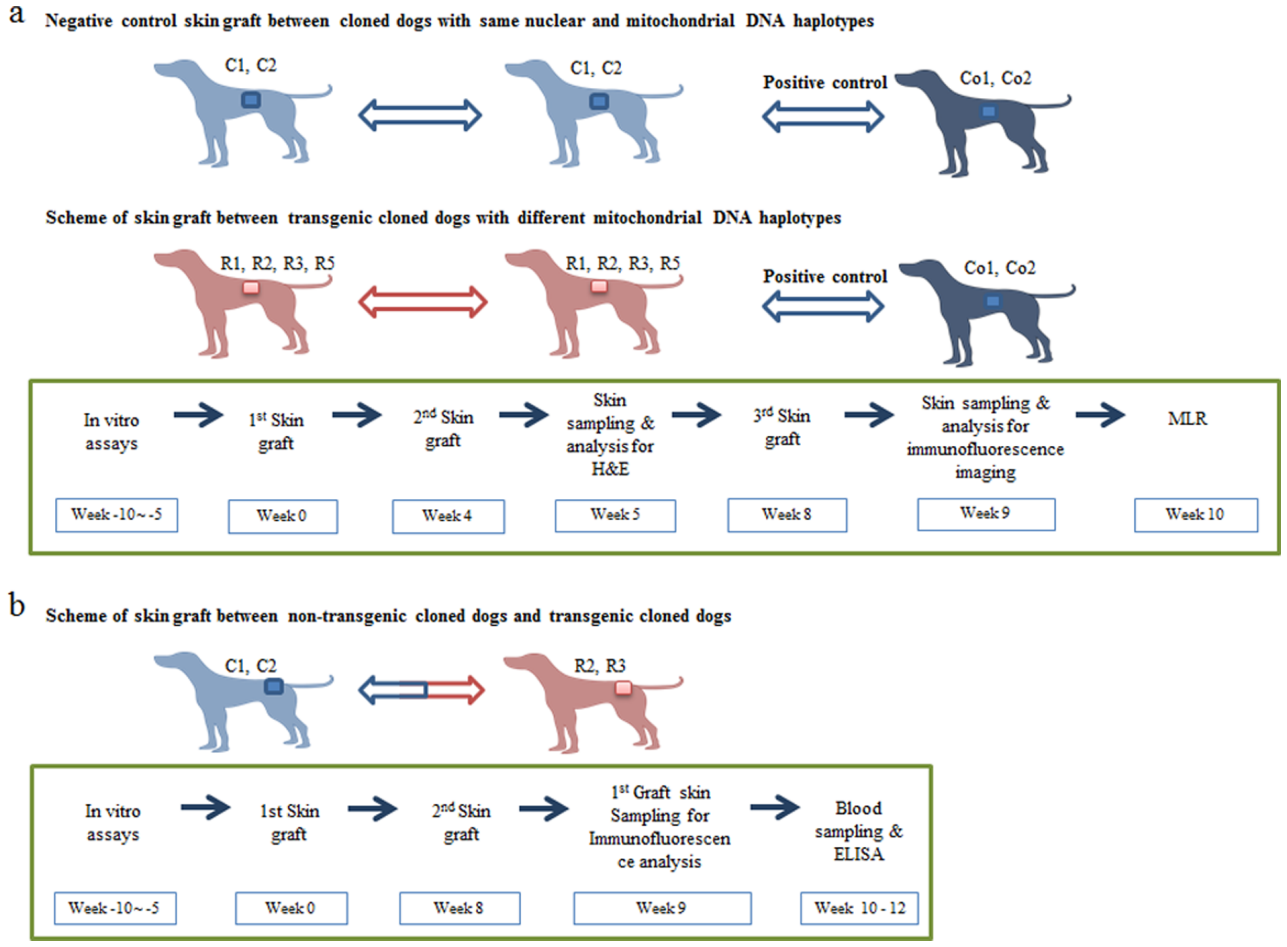
Experimental design and image analysis result between cloned dogs. (a) Experimental design and timeline of skin graft between cloned dogs with different mitochondrial haplotypes. As negative control, auto grafts as well as cloned dogs with same mtDNA haplotype (C1, C2) were used. Before skin graft, all *in vitro* assays were performed. For H&E staining, immunofluorescence imaging, 1^st^ skin graft fragments were analyzed. (b) Experimental design and timeline between transgenic cloned dogs and non-transgenic cloned dogs. Before skin graft, all *in vitro* assays were performed. All dogs were tested twice for each skin graft, then skin samplings were performed. For immunofluorescence imaging, 1^st^ skin graft fragments were analyzed. RFP expression were monitored until 63 days after skin graft.

**Figure 2:**
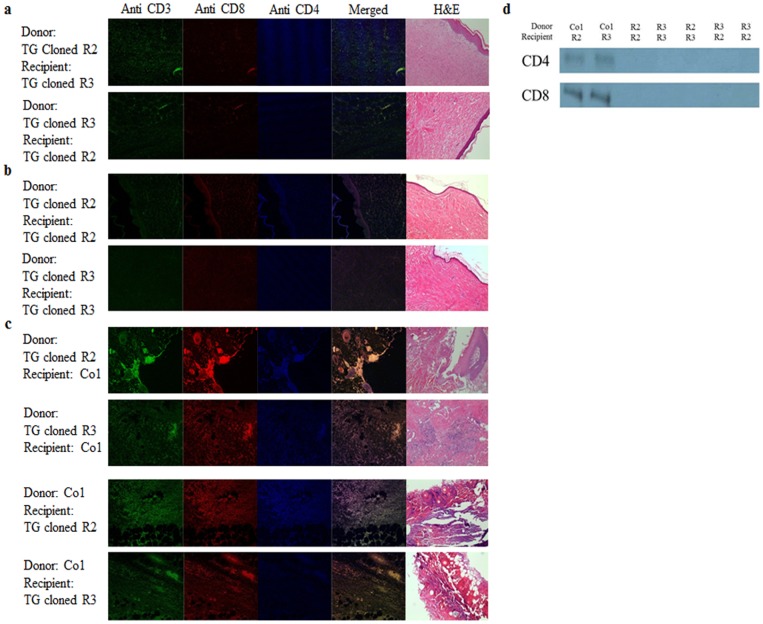
Absence of *in vivo* immunogenicity in skin grafts between cloned dogs with different mitochondrial DNA sequences. No evidence with infiltration of T cell was detected in the skin segments transplanted into the recipient dogs with different mitochondrial haplotypes (a). Sections from skin segments of autografts were used as negative controls (b). Sections from skin segments of cloned dogs transplanted into control dogs were used as positive controls (c). Western blot analysis confirms high protein levels of CD4 and CD8 in the positive controls, whereas CD4 and CD8 expression intensities were significantly lower in allograft of cloned dogs with different mitochondrial haplotypes (d). Upper lane indicates the donor dog and lower lane means recipient dog.

To gain insights into the therapeutic applicability of canine skin tissues with different mtDNA haplotypes, skin grafting between cloned dogs was performed to determine immunological compatibility *in vivo* (Fig. 1). Whereas allogeneic Co 1 (non-related control dogs) skin fragments were rapidly rejected in R2 (transgenic cloned dog) and R3 (transgenic cloned dog) recipients with massive infiltration of CD4+, CD8+ T cells, infiltration, edema and perivascular inflammation 7 days after 2^nd^ skin graft, skin tissues of R2 and R3 were accepted in R3 and R2 recipients as well as autografts, without any evidence of immune rejection (Fig. 2). Likewise, skin segments from cloned dogs with different mtDNA sequences did not induce immune rejection in the recipient cloned dogs (Fig. S2). In MLR of 10 weeks after 3^rd^ skin graft, we couldn’t detect any sign of mtDNA derived minor antigen immunogenicity with no significant differences compared to those of MLR before skin graft (data not shown).

In mice, mtDNA encoded proteins could elicit rejection by innate immunity in a setting where the genomic DNA matched [24], [25]. Furthermore, kidneys transplanted between cloned pigs differing in some mtDNA genes rejected those grafts [6]. Therefore, different antigenicity of grafts from different tissues could be also considered. In our experiment, despite a high level of diversity of mtDNA haplotypes heteroplasmy among domestic dogs [26], skin grafts were successfully accepted in at least 20 donor-recipient combinations. In cattle and pigs, it was shown that SCNT-derived tissues were not rejected by the immune system of the nucleus donor after SCNT in skin graft [7], [27], [28]. Our findings suggest that differences of canine mtDNA haplotypes could not elicit skin graft rejection among cloned dogs, as previously observed in cattle and pigs.

**Figure 3:**
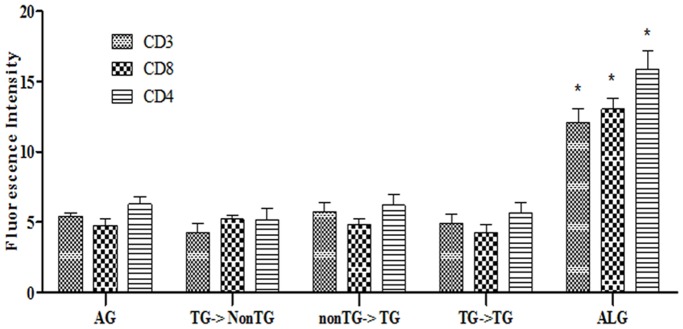
Expression levels of CD3, CD4 and CD8 of skin grafts between cloned dogs using fluorescence image analysis. Immunological response level of CD3, CD4 and CD8 were similar in AG (autograft), TG-> NonTG (donor: transgenic dog, recipient: non-transgenic cloned dogs), NonTG->TG (donor: non-transgenic dog, recipient: transgenic cloned dogs) and TG cloned dogs (donor: transgenic dog, recipient: transgenic cloned dogs). However, Both of ALG (allograft) between TG dogs and non-related control dogs and allograft between non-TG dogs and non-related control dogs shows significantly higher intensity of immunological response (p<0.05). Results are presented as mean ± SEM. Replication number is at least 8 times.

We also showed genetic identity between tissues of non-transgenic cloned dogs (C1, C2) derived from beagle fibroblasts (BF3) [9] and tissues of transgenic cloned dogs (R1, R2, R3 and R5) derived from BF3 transfected with RFP (Table S1.) [2]. Immunological compatibility between these dogs was completely established through *in vitro* tests such as DLA typing and MLR (Fig. S1). Skin tissues of non-transgenic cloned dogs were transplanted into transgenic cloned dogs and *vice-versa*. Skin tissues derived from cloned dogs were transplanted with no immune rejection, as determined by T cell infiltration of peri-graft skin sections after 7 days of 2^nd^ skin graft. Despite insertion of the foreign gene RFP in transgenic cloned dogs, skin tissue from RFP transgenic cloned dogs was completely accepted in non-transgenic cloned dog recipients (Fig. 3, Fig. S3). These finding indicate that foreign gene insertion in cloned dogs did not induce a T cell-dependent skin graft rejection response in syngeneic recipients. It has been suggested that the nuclear reprogramming process in SCNT could result in surface expression of proteins and molecules unknown to the immune system of the graft recipients. In this regard, in inbred mice, enhanced GFP (eGFP) skin transplantation causes an acute reaction [29]. It was proved that eGFP also induce immune responses that interfere with its applicability in gene insertion of mouse [30]. However, our results suggest that inserted foreign gene, RFP has no immunological effects on the antigens of transgenic cloned dogs against to the non-transgenic cloned dogs. It also suggested that non-transgenic cloned dogs produced by SCNT using transfected cells have no immune regulatory effect on the host immune system and that the canine SCNT process did not result in surface expression of immunogenic molecules. Nonetheless, the possibility of immune rejection of other foreign genes, for example, pathogenically relevant transgene in clinical science remains to be confirmed.

**Figure 4:**
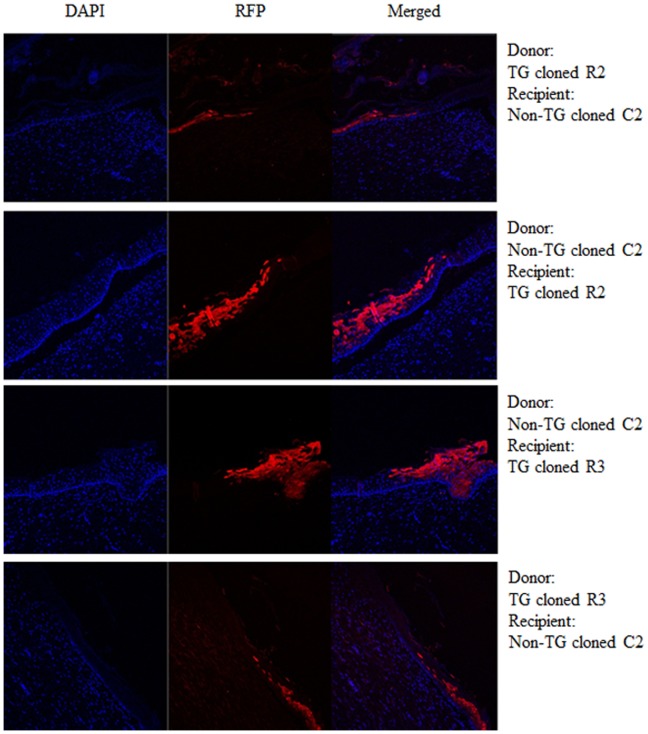
Maintenance of foreign gene expression between transgenic cloned beagle with foreign genes and non-transgenic cloned dogs. No expression of foreign gene in non-transgenic dog (C2) recipient was maintained in skin graft of transgenic cloned dog (R2). The limit between donor and recipient were not changed until 63 days after skin graft.

Finally we examined whether functional expression of RFP was maintained in skin tissue grafts. During the course of this experiment, the expression level of RFP positive skin tissues were maintained for at least 63 days after surgery and RFP positive cells were detected in the epidermis, hair follicles and sebaceous glands (Fig. 4 and Table S1). It has been suggested that the co-expression of selection markers can limit or abrogate the persistence of expression of therapeutic genes [31], [32]. The potential success of gene therapy or production of transgenic cloned dogs may depend on long-term transgene expression to cure or slow down the progression of disease. In addition, there were no host immune responses to the skin grafts among transgenic dogs and non-transgenic cloned dogs, and it appears that the level and duration of RFP transgene expression was not affected. This also indicates possible successful of therapeutic transplantation of tissues or cells derived from transgenic cloned dogs. In addition, the insertion site of the RFP gene into genomic DNA is not the same in all experimental dogs (Table S2). If the RFP gene insertion site can affect the immune response, it should affect the results of syngeneic skin grafting. However, no immune rejection was apparent in skin grafts with different transgene insertion sites. Our findings indicate that SCNT-derived somatic cells with or without foreign genes can be accepted in syngeneic recipients.

Our study established that tissues derived from canine SCNT can be accepted in syngeneic recipients despite different mtDNA haplotypes. We also provide evidence that skin segments containing a foreign gene are sufficiently acceptable to syngeneic recipients with or without the foreign gene. Taken together, these data indicate that SCNT using transgenic technology can support immunological compatibility between genetically engineered tissues and patients and thereby help to accelerate clinical therapeutic research and its applications.

## Supporting Information

Figure S1 **Immunological feature of transgenic dogs and non-transgenic dogs.** (a) Molecular typing of dog leukocyte antigen, DLA-88 (MHC class I), DRB, DQA1, DQB1(MHC class II) polymorphic region in all cloned dogs (C1, C2, R1, R2, R3, and R5). (b) *In vitro* immunogenicity test using mixed lymphocyte reaction between all experimental dogs before skin graft. (c) Blood typing. (d) Analysis of blood crossmatching in all cloned dogs and control dogs.
(PDF)

Figure S2 **Fluorescence image analysis of skin grafts between cloned dogs with different mtDNA haplotypes**
(PDF)

Figure S3 **Absence of** ***in vivo*** **immune rejection between non-transgenic dogs and transgenic dogs.** (a) Positive control of skin graft, as donor skin segments were derived from non-related control dogs (Co1, Co2), they were completely rejected in the graft bed in transgenic cloned dogs (R2, R3). (b) However, skin grafts between a transgenic cloned dog, R2 and a non-transgenic cloned dog, C1 showed no apparent immune rejection. Similarly, as shown in (c) R2 - C2, (d) R3-C1, (e) R3-C2, there was no immune rejection in these grafts as well. (f) Western blot analysis of the skin graft between cloned dogs confirmed the expression of CD4 and CD8 protein only in the graft between cloned dogs and non-related control dogs.
(PDF)

Figure S4 **Foreign gene expression between skin graft of two transgenic dogs (R2, R3).** Red fluorescent protein expression in skin graft was maintained after 63 days skin graft in syngenic graft beds.
(PDF)

Table S1 **Genetic background for microsatellite analysis of two non-transgenic cloned dogs and four transgenic cloned dogs.**
(PDF)

Table S2 **Insertion site of foreign gene, RFP in transgenic cloned dogs.**
(PDF)
