# Supplementary figures and images for: Survival of Skin Graft between Transgenic Cloned Dogs and Non-Transgenic Cloned Dogs
Source: PLoS One. 2014 Nov 5;9(11):e108330. doi: 10.1371/journal.pone.0108330 (PMC4220905; doi:10.1371/journal.pone.0108330)

Figure S1.

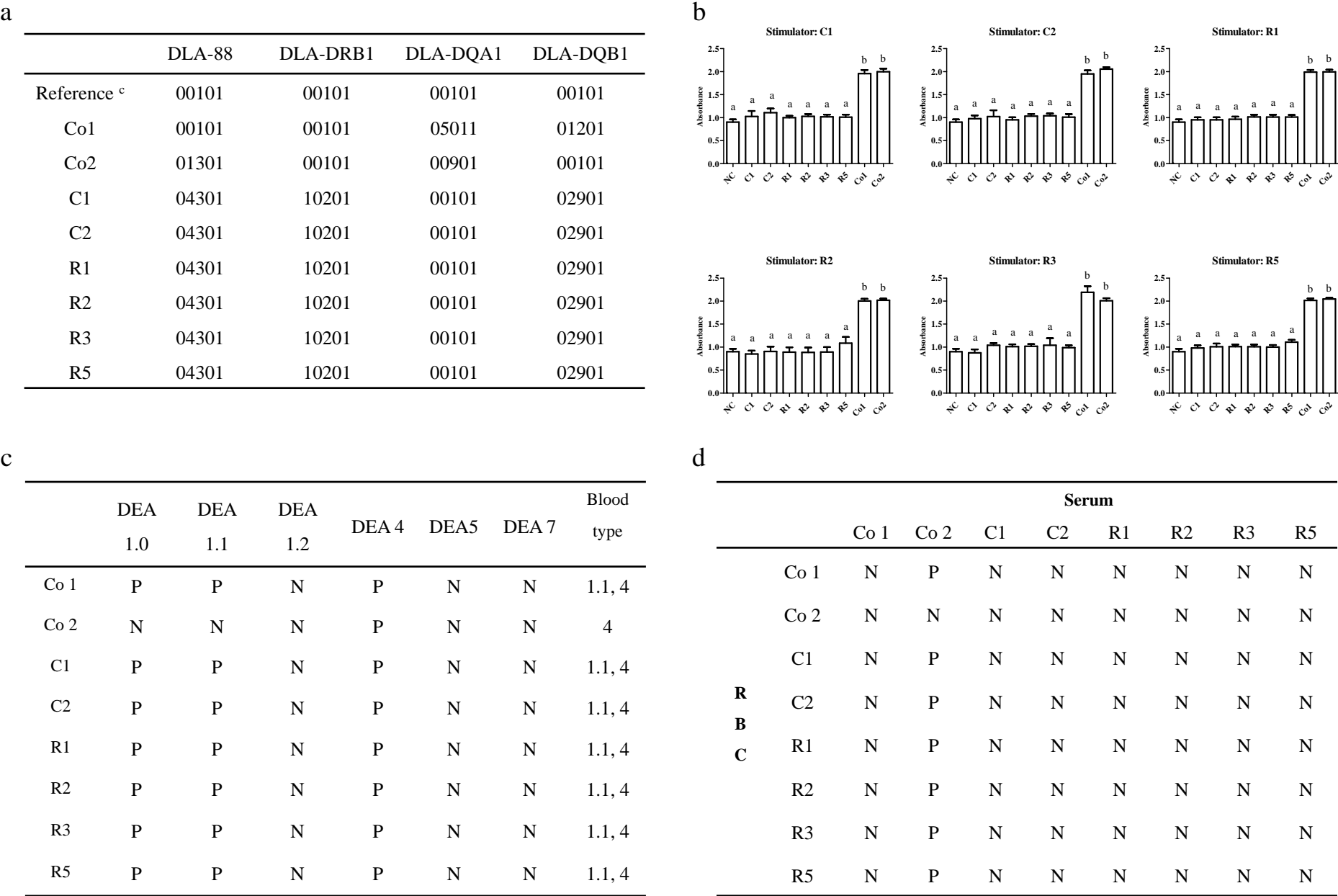

Supplement: Figure S1 — Immunological feature of transgenic dogs and non-transgenic dogs. (a) Molecular typing of dog leukocyte antigen, DLA-88 (MHC class I), DRB, DQA1, DQB1(MHC class II) polymorphic region in all cloned dogs (C1, C2, R1, R2, R3, and R5). (b) In vitro immunogenicity test using mixed lymphocyte reaction between all experimental dogs before skin graft. (c) Blood typing. (d) Analysis of blood crossmatching in all cloned dogs and control dogs. (PDF) [file pone.0108330.s001.pdf]

Figure S2.

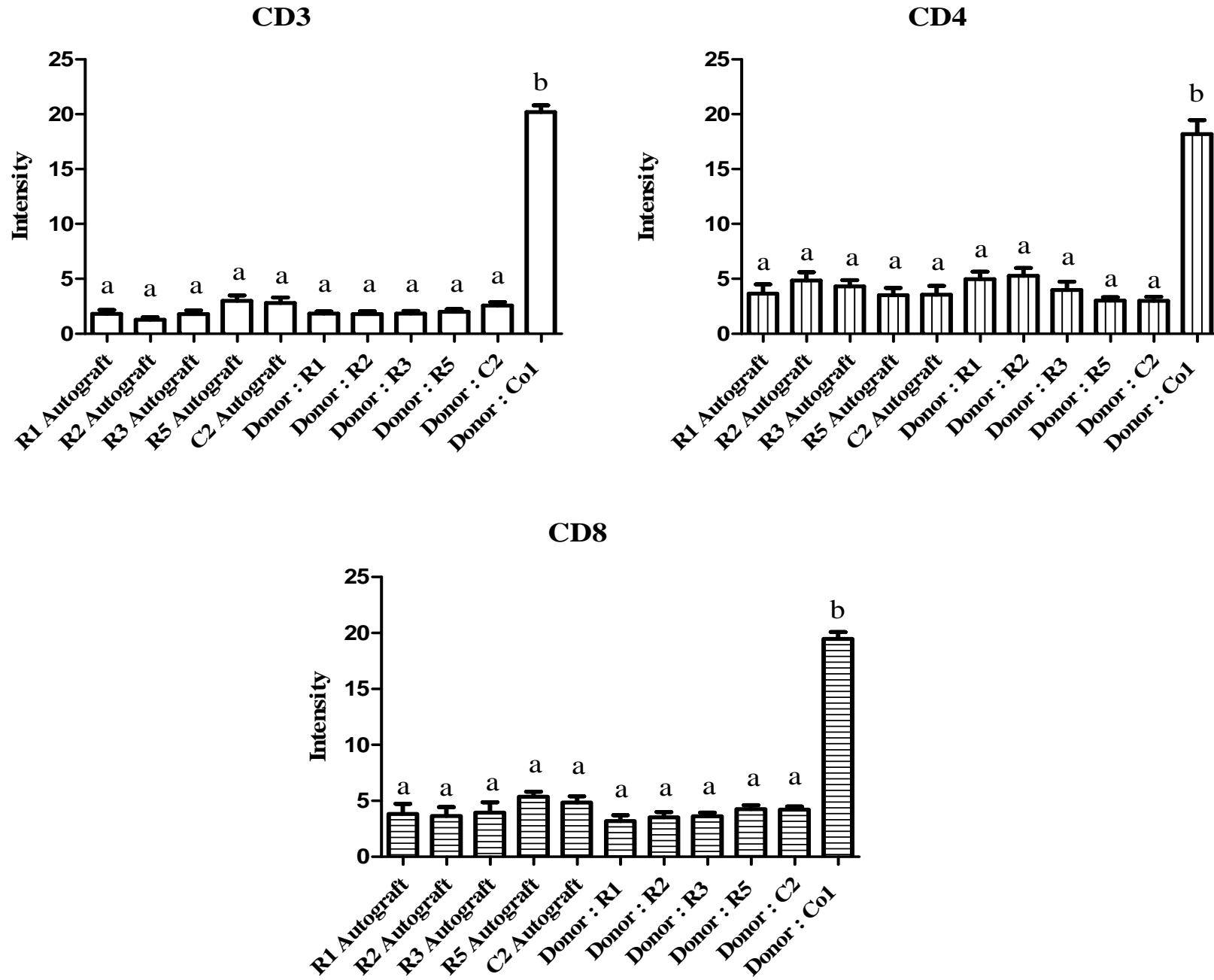

Supplement: Figure S2 — Fluorescence image analysis of skin grafts between cloned dogs with different mtDNA haplotypes (PDF) [file pone.0108330.s002.pdf]

**Figure S3.**

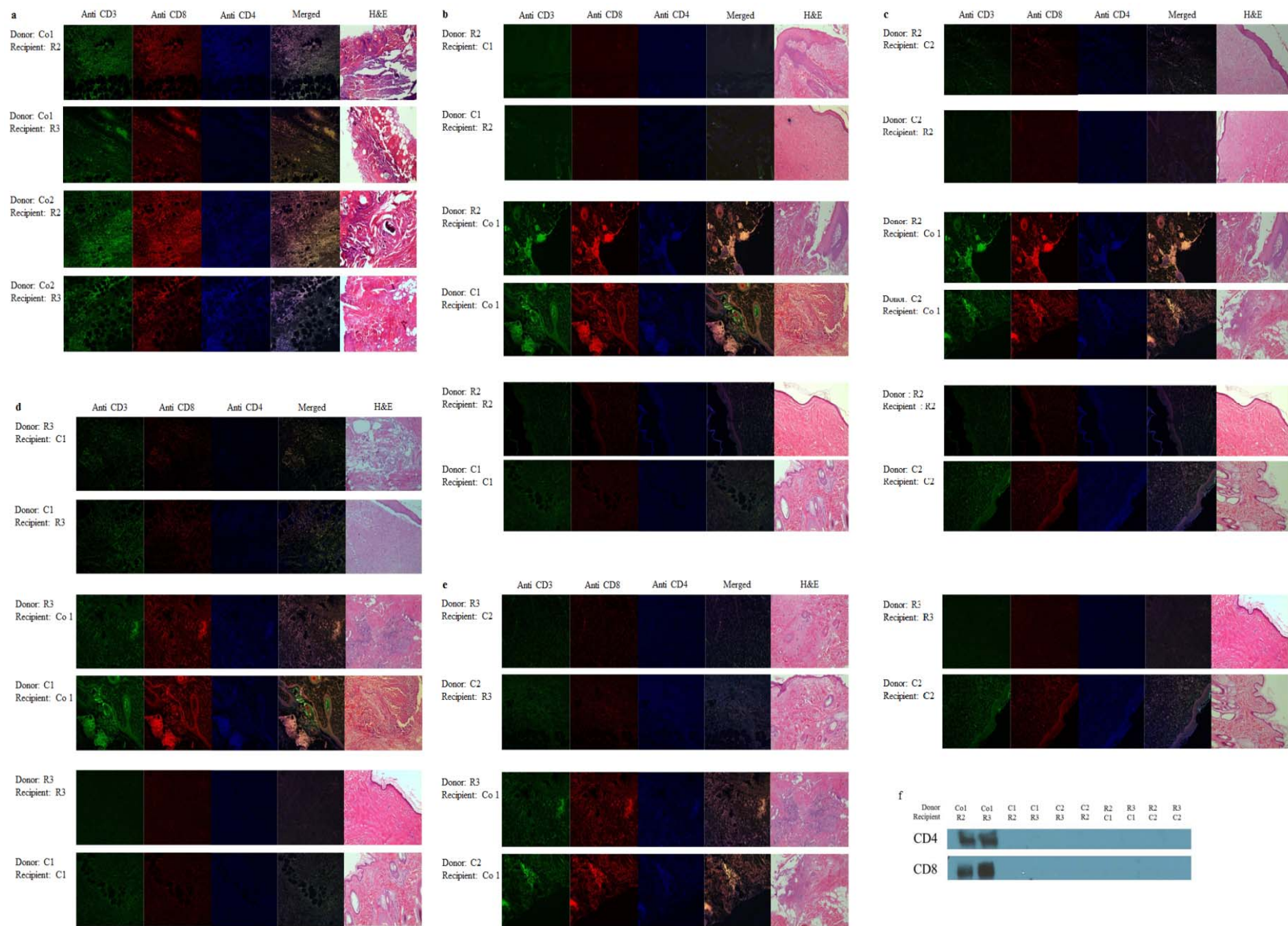

Supplement: Figure S3 — Absence of in vivo immune rejection between non-transgenic dogs and transgenic dogs. (a) Positive control of skin graft, as donor skin segments were derived from non-related control dogs (Co1, Co2), they were completely rejected in the graft bed in transgenic cloned dogs (R2, R3). (b) However, skin grafts between a transgenic cloned dog, R2 and a non-transgenic cloned dog, C1 showed no apparent immune rejection. Similarly, as shown in (c) R2 - C2, (d) R3-C1, (e) R3-C2, there was no immune rejection in these grafts as well. (f) Western blot analysis of the skin graft between cloned dogs confirmed the expression of CD4 and CD8 protein only in the graft between cloned dogs and non-related control dogs. (PDF) [file pone.0108330.s003.pdf]

**Figure S4.**

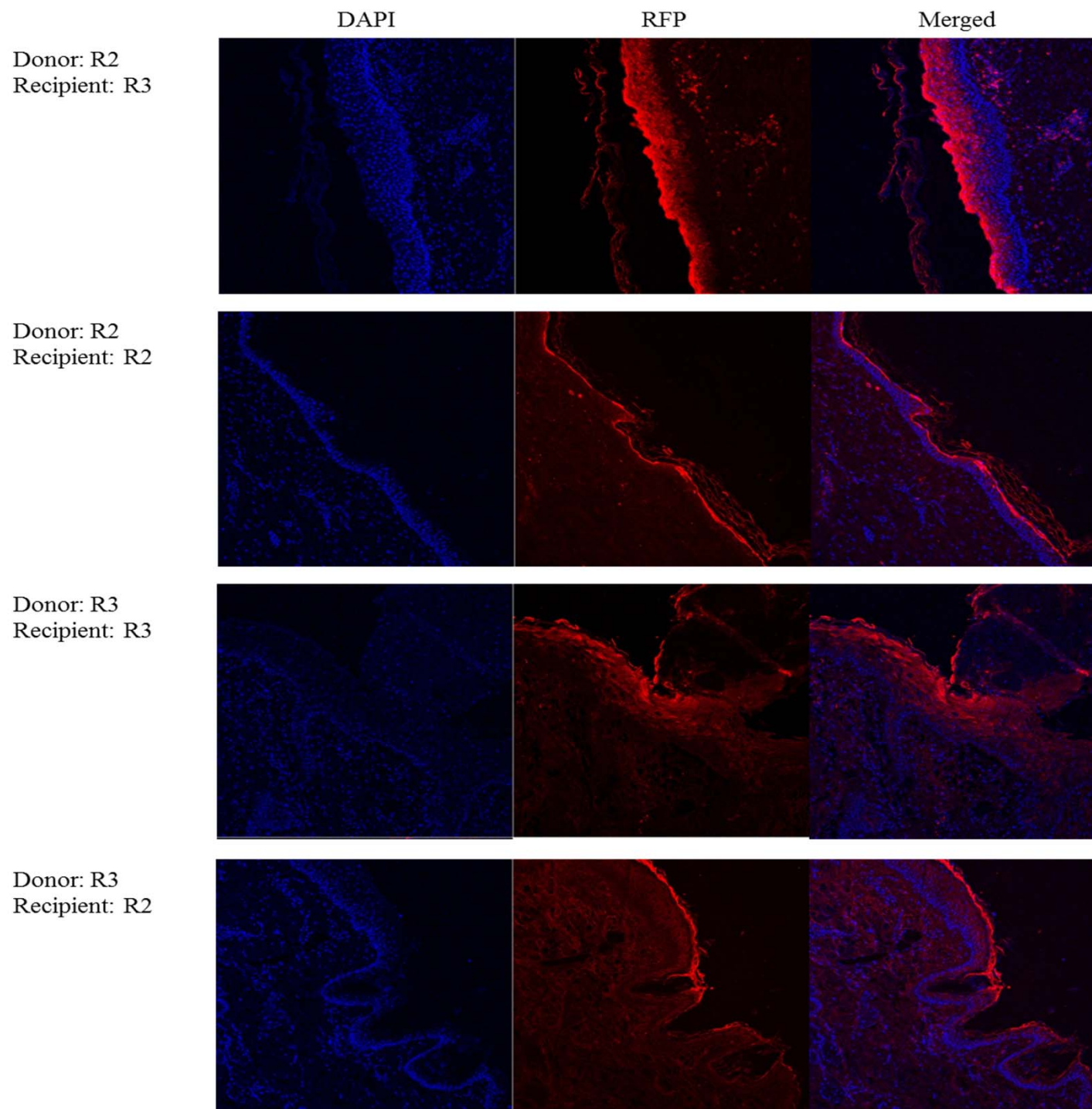

Supplement: Figure S4 — Foreign gene expression between skin graft of two transgenic dogs (R2, R3). Red fluorescent protein expression in skin graft was maintained after 63 days skin graft in syngenic graft beds. (PDF) [file pone.0108330.s004.pdf]
