# Supplementary material for: Survival of Skin Graft between Transgenic Cloned Dogs and Non-Transgenic Cloned Dogs
Source: PLoS One. 2014 Nov 5;9(11):e108330. doi: 10.1371/journal.pone.0108330 (PMC4220905; doi:10.1371/journal.pone.0108330)
Supplement: Table S1 — Genetic background for microsatellite analysis of two non-transgenic cloned dogs and four transgenic cloned dogs. (PDF) [file pone.0108330.s005.pdf]

Table S1.

Marker	PEZ 01	PEZ 02	PEZ 05	PEZ 17	FH 2010	FH 2054	FH 2079
Non-TG somatic cells BF3	114/114	127/127	101/105	211/211	228/232	147/155	273/273
Non-TG cloned C1	114/114	127/127	101/105	211/211	228/232	147/155	273/273
Non-TG cloned C2	114/114	127/127	101/105	211/211	228/232	147/155	273/273
TG somatic cells	114/114	127/127	101/105	211/211	228/232	147/155	273/273
TG cloned R1	114/114	127/127	101/105	211/211	228/232	147/155	273/273
TG cloned R2	114/114	127/127	101/105	211/211	228/232	147/155	273/273
TG cloned R3	114/114	127/127	101/105	211/211	228/232	147/155	273/273
TG cloned R5	114/114	127/127	101/105	211/211	228/232	147/155	273/273
