# Supplementary material for: Survival of Skin Graft between Transgenic Cloned Dogs and Non-Transgenic Cloned Dogs
Source: PLoS One. 2014 Nov 5;9(11):e108330. doi: 10.1371/journal.pone.0108330 (PMC4220905; doi:10.1371/journal.pone.0108330)
Supplement: Table S2 — Insertion site of foreign gene, RFP in transgenic cloned dogs. (PDF) [file pone.0108330.s006.pdf]

Table S2.

ID	Chromosome No.	Location
TG cloned R1	25	5 M bp – 6Mbp intron
TG cloned R2	25	47.22Mbp - 47.23Mbp intron
TG cloned R3	2	34.24-34.25Mbp intron
TG cloned R5	2	34.24-34.25Mbp intron
